# Correlation between plasma proprotein convertase subtilisin/kexin type 9 and blood lipids in patients with newly diagnosed primary nephrotic syndrome

**DOI:** 10.1080/0886022X.2020.1756846

**Published:** 2020-04-29

**Authors:** Huaying Shen, Sheng Feng, Ying Lu, Linsen Jiang, Tingting Yang, Zhi Wang

**Affiliations:** Department of Nephrology, The Second Affiliated Hospital of Soochow University, Suzhou, China

**Keywords:** Nephrotic syndrome, hypercholesterolemia, PCSK9, cholesterol, triglycerides, proteinuria

## Abstract

**Background:**

Proprotein convertase subtilisin/kexin type 9 (PCSK9) is a major post-transcriptional regulator of low-density lipoprotein receptor degradation. Recently, PCSK9 was shown to be overexpressed by liver cells in rats with proteinuria. However, the levels of PCSK9 in newly diagnosed primary nephrotic syndrome (PNS) patients and correlations involving PCSK9 and blood lipids are not clearly understood.

**Methods:**

One hundred and sixteen patients who were newly diagnosed with PNS were enrolled in this study.

**Results:**

Plasma PCSK9 levels in PNS patients were significantly higher than those in healthy controls [310.86 (250.87, 390.25) ng/ml vs 255.67 (202.26, 320.26) ng/ml, *p* = 0.002]. Plasma PCSK9 in PNS patients was positively correlated with total cholesterol (TC) and low-density lipoprotein cholesterol (LDL-C) (γ = 0.246, *p* = 0.008, and *γ* = 0.183, *p* = 0.049). When plasma PCSK9 was >267.60 ng/ml, the risk of developing hypercholesterolemia significantly increased in PNS patients (OR = 6.40, 95% CI 2.06–19.87, *p* = 0.001). When plasma PCSK9 was >255.05 ng/ml, the risk of developing higher levels of LDL-C significantly increased in PNS patients (OR = 3.83, 95%CI 1.25–11.68, *p* = 0.018).

**Conclusions:**

Plasma PCSK9 levels in newly diagnosed PNS patients were markedly increased, and elevated PCSK9 abundance was positively correlated with elevated serum TC and LDL-C levels, suggesting that PCSK9 may emerge as a novel therapeutic target in NS-associated hypercholesterolemia.

## Introduction

Nephrotic syndrome (NS) is one of the most common clinical syndromes in terms of glomerular diseases. It is defined by proteinuria >3.5 g/day and serum albumin <3.0 g/dL [1]. Hypercholesterolemia and hypertriglyceridemia are seen in 90% and 78% of NS cases, respectively, with a combined prevalence of 74% in the nephrotic population [2,3]. The mechanisms underlying hypercholesterolemia in NS patients involve abnormalities in apolipoprotein synthesis, and lower fractional catabolic rate of low-density lipoprotein cholesterol (LDL-C) due to reduction in low-density lipoprotein receptor (LDL-R) levels [2,4,5]. Available data suggest that the latter mechanism is more important [3,6]. Persistent hypercholesterolemia may accelerate glomerulosclerosis through a variety of mechanisms [7,8] and predispose patients to atherosclerotic cardiovascular (CV) disease [9,10]. Lowering LDL-C using statins and/or ezetimibe represents the gold standard of lipid-lowering therapy, with a great body of evidence from several large clinical trials. Although statins have the abovementioned advantages, in some patients using the maximum tolerated dose of statins, plasma cholesterol still does not meet relevant standards.

Proprotein convertase subtilisin/kexin type 9 (PCSK9) is a member of the serine protease superfamily and is a major post-transcriptional regulator of LDL-R degradation [11–14]. Recently, PCSK9 was shown to be overexpressed by liver cells in an NS model [11]. Therefore, PCSK9 may play a critical role in LDL-C metabolism in patients with NS [15].

In this study, 116 patients with newly diagnosed primary NS (PNS) were enrolled, and differences in plasma PCSK9 levels between PNS patients and a healthy control group were evaluated. Correlations between PCSK9 and traditional blood lipid indicators were also investigated.

## Materials and methods

### Patients

Patients who were diagnosed with PNS in the Department of Nephrology, Second Affiliated Hospital of Soochow University, China, between June 2015 and December 2018 were enrolled in the study. Inclusion criteria were patients diagnosed with PNS based on clinical manifestations and laboratory tests. The exclusion criteria were as follows: (1) patients with secondary NS; (2) patients with diabetes mellitus, hepatitis, or liver cirrhosis; (3) patients with a body mass index (BMI) ≥28 kg/m^2^; (4) patients receiving lipid-lowering drugs for long-term treatment; and (5) patients undergoing steroid therapy and (or) immunosuppressive therapy. In total, 116 patients with newly diagnosed PNS were recruited. In addition, 30 healthy age- and sex-matched individuals were selected as a control group. Two milliliters of fasting plasma samples from the enrolled patients was collected in the morning on the day of renal biopsy.

### Clinical data collection

Demographic, clinical, and laboratory data of the NS patients were collected and recorded, including age, gender, total cholesterol (TC), triglycerides (TG), LDL-C, very low-density lipoprotein cholesterol (VLDL-C), high-density lipoprotein cholesterol (HDL-C), 24 h urine protein, plasma albumin (ALB), creatinine (Cr), blood urea nitrogen (BUN), estimated glomerular filtration rate (eGFR), alanine aminotransferase (ALT), aspartate aminotransferase (AST), hemoglobin (HGB), blood glucose, and uric acid (UA). All laboratory tests were performed at the Laboratory of the Second Affiliated Hospital of Soochow University. We used the Chronic Kidney Disease Epidemiology Collaboration (CKD-EPI) creatinine equation to estimate the GFR of the enrolled subjects [16].

### Plasma PCSK9 assay

Plasma PCSK9 levels were determined using a double-antibody sandwich, avidin-biotin-horseradish peroxidase enzyme complex, and enzyme-linked immunosorbent assay (ABC-ELISA). An anti-human PCSK9 monoclonal antibody (R & D Systems, USA) was used to coat the ELISA plate. PCSK9 in standard and test samples became bound to the monoclonal antibody. Unbound reagents were washed away, and biotinylated anti-human PCSK9 antibody was added, forming an immune complex adherent to the plate. Horseradish peroxidase-labeled streptavidin binds to biotin. If PCSK9 was present in the reaction well, the chromogenic substrate exhibited a blue color and then became yellow after adding a stop solution. The optical absorbance was measured at 450 nm.

### Statistical analysis

SPSS 23.0 software was used for data analysis. Normally distributed measurement data are presented as $\bar{\chi }\;$± s, and non-normally distributed data are presented as *M* (1/4, 3/4). Enumeration data are expressed as the number of cases and percentage. Normally distributed measurement data were compared between two groups using Student’s *t*-test, while comparisons among multiple groups were performed using analysis of variance (ANOVA). The Mann-Whitney *U* test was used to compare non-normally distributed data between two groups and among multiple groups. Correlations between two continuous variables with normal distribution was analyzed by Pearson’s correlation analysis, and correlations involving non-normally distributed data was performed by Spearman rank-order correlation analysis. The effect of elevated plasma PCSK9 levels on the risk of developing hyperlipidemia in patients with NS was analyzed using binary logistic regression. *p* < 0.05 was considered statistically significant.

## Results

### Clinical characteristics of patients with PNS and healthy controls

Of the 116 newly diagnosed PNS patients, 66 were male (56.9%). The average age of the patients at the time of renal biopsy was 48.32 ± 14.03 years. Plasma levels of TC, LDL-C, TG, VLDL-C, Cr, BUN, and UA were significantly higher and ALB and eGFR levels were significantly lower in the PNS patients compared with those in the healthy controls (*p* < 0.05). There were no significant differences in age or gender between the two groups (Table 1).

**Table 1.** Comparison of clinical characteristics of PNS patients and healthy controls.

Characteristic	PNS (*n* = 116)	Healthy Controls (*n* = 30)	*p* Value
Age (years)	48.32 ± 14.03	45.60 ± 6.19	0.118
Male, *n* (%)	66 (56.9)	19 (63.3)	0.678
TC (mmol/l)	8.28 ± 2.42	4.59 ± 0.49	<0.001
lDl-C (mmol/l)	5.70 ± 2.25	2.46 ± 0.41	<0.001
TG (mmol/l)	2.08 (1.42, 3.12)	0.70 (0.57, 0.84)	<0.001
HDl-C (mmol/l)	1.40 (1.09, 1.88)	1.52 (1.33, 1.80)	0.203
VlDl-C (mmol/l)	0.82 (0.64, 1.17)	0.60 (0.52, 0.69)	<0.001
AlB (g/l)	23.06 ± 5.46	45.49 ± 2.15	<0.001
AlT (U/l)	16.00 (11.00, 23.00)	13.00 (9.75, 16.00)	0.009
AST (U/l)	21.00 (17.00, 25.75)	16.00 (14.00, 18.00)	<0.001
Scr (umol/l)	71.00 (59.25, 87.50)	54.50 (46.75, 61.50)	<0.001
BUN (mmol/l)	5.15 (4.23, 7.25)	4.50 (3.78, 5.30）	0.010
UA (umol/l)	358.00 (291.00, 427.50)	238.50 (204.00, 281.00)	<0.001
eGFR (ml/min/1.73m^2^)	99.50 (76.25, 112.00)	115.00 (107.75, 122.50)	<0.001
HGB (g/l)	133.34 ± 21.07	135.83 ± 13.15	0.423

Abbreviations: PNS: primary nephrotic syndrome; TC: total cholesterol; LDL-C: low-density lipoprotein cholesterol; TG: triglycerides; HDL-C: high-density lipoprotein cholesterol; VLDL-C: very low-density lipoprotein cholesterol; ALB: albumin; ALT: alanine aminotransferase; AST: aspartate aminotransferase; Scr: serum creatinine; BUN: blood urea nitrogen; UA: uric acid; eGFR: estimated glomerular filtration rate; HGB: hemoglobin.

### Plasma PCSK9 levels in patients with PNS

**Figure 1.**
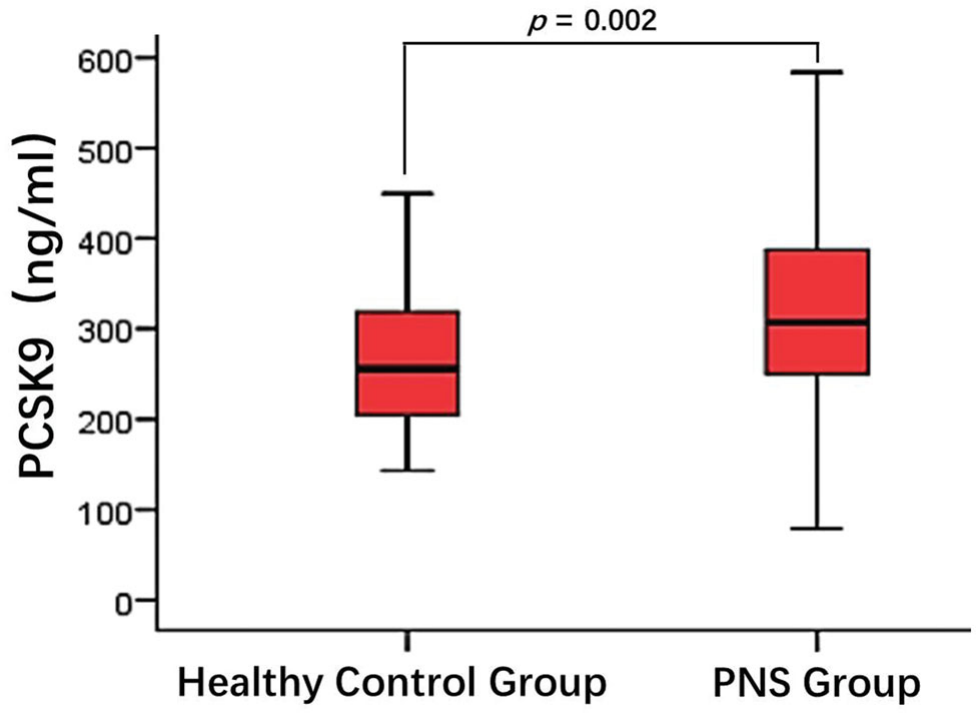
Comparison of plasma PCSK9 between patients with PNS and healthy controls. The plasma PCSK9 levels in patients with PNS were significantly higher than that in the healthy controls (*p* = 0.002).

ELISA results showed that plasma PCSK9 levels in PNS patients were significantly higher than in healthy controls [310.86 (250.87, 390.25) ng/ml vs. 255.67 (202.26, 320.26) ng/ml]. This difference was statistically significant (*p* = 0.002, Figure 1).

### Correlation between plasma PCSK9 and blood lipids in patients with PNS

**Figure 2.**
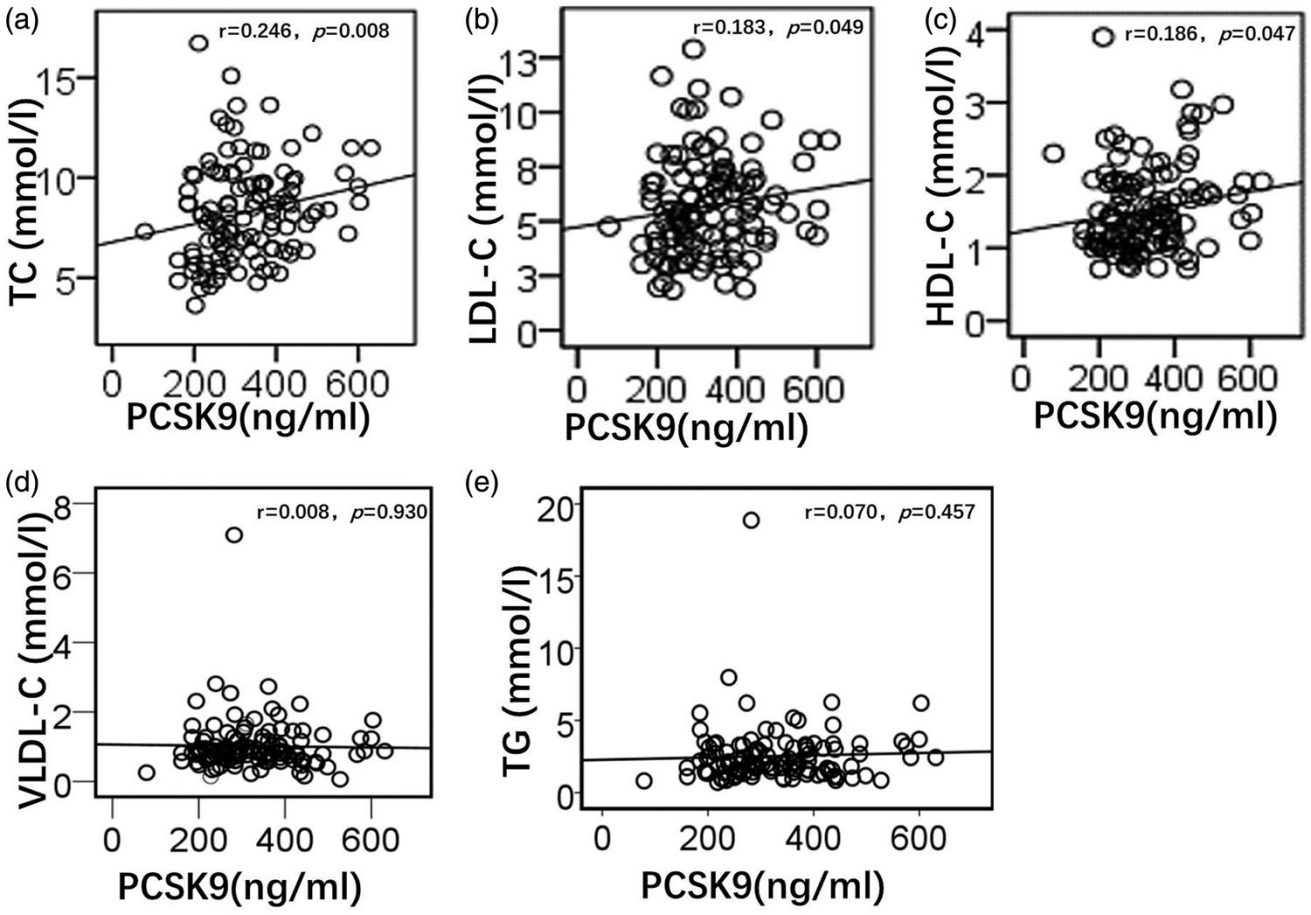
Correlation analysis of plasma PCSK9 and blood lipids in patients with PNS. Plasma PCSK9 levels in patients with PNS had a positive linear correlation with TC (a), LDL-C (b), and HDL-C (c). Plasma PCSK9 levels in patients with PNS had no linear correlation with VLDL-C (d) and TG (e). TC, total cholesterol; LDL-C, low-density lipoprotein cholesterol; HDL-C, high-density lipoprotein cholesterol; VLDL-C, very low-density lipoprotein cholesterol; TG, triglycerides.

Spearman correlation analysis showed that plasma PCSK9 levels in patients with PNS had positive linear correlations with TC *(r* = 0.246*, p* = 0.008), LDL-C (*r* = 0.183, *p* = 0.049), and HDL-C (*r* = 0.186, *p* = 0.047), whereas there was no linear correlation with VLDL-C (*r* = 0.008, *p* = 0.930) and TG (*r* = 0.070, *p* = 0.457) (Figure 2).

### Correlation of plasma PCSK9 with proteinuria and plasma ALB in patients with PNS

**Figure 3.**
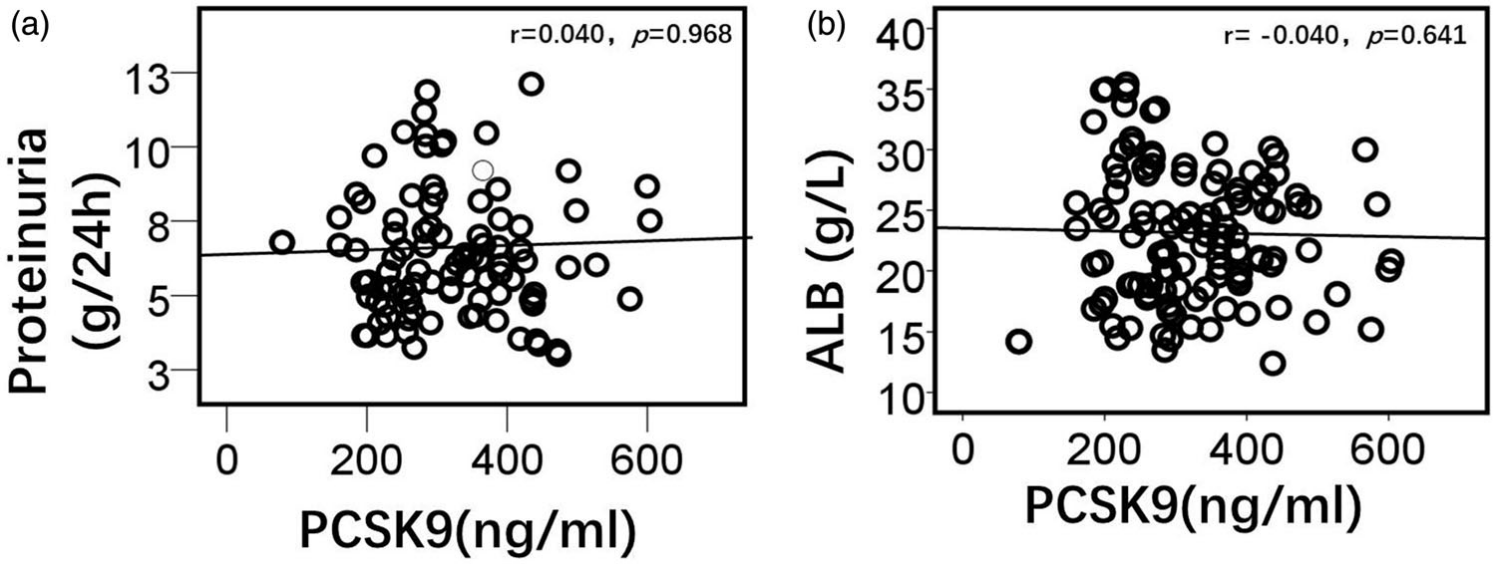
Correlation analysis of plasma PCSK9 with proteinuria and plasma albumin in patients with PNS. Plasma PCSK9 levels in patients with PNS had no linear correlation with the amount of proteinuria (a) or plasma albumin (b). ALB, albumin.

Spearman correlation analysis showed that plasma PCSK9 levels in patients with PNS had no linear correlation with the amount of proteinuria per 24 h *(r* = 0.040, *p* = 0.968) or plasma ALB (*r* = −0.040, *p* = 0.641) (Figure 3).

### Effects of plasma PCSK9 levels on hyperlipidemia in PNS patients

**Figure 4.**
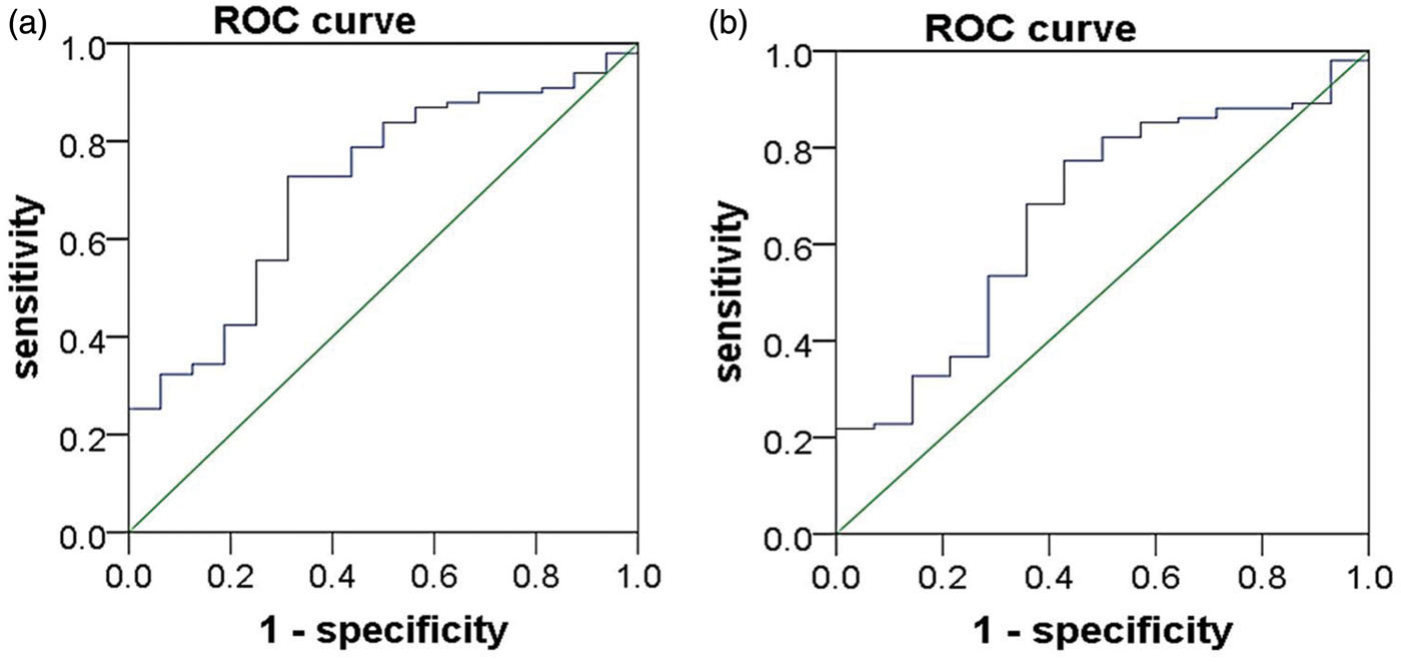
ROC curve of plasma PCSK9 in the prediction of hyperlipidemia in patients with PNS. (a)The area under the receiver operating characteristic (ROC) curve (AUC) for the prediction of hypercholesterolemia in NS patients using plasma PCSK9 was 0.71 (95% CI 0.58–0.84, *p* = 0.007). (b) The AUC for the prediction of high levels of LDL-C in NS patients using plasma PCSK9 was 0.66 (95% CI 0.52–0.81, *p* = 0.047).

The area under the receiver operating characteristic (ROC) curve (AUC) for the prediction of hypercholesterolemia in NS patients using plasma PCSK9 was 0.71 (95% CI 0.58–0.84, *p* = 0.007) (Figure 4(a)), the Youden index was 0.42, and the corresponding PCSK9 value was 267.60 ng/ml. The sensitivity of PCSK9 in predicting hypercholesterolemia was 0.73, and the specificity was 0.69. Logistic regression analysis showed that when plasma PCSK9 was >267.60 ng/ml, the risk of developing hypercholesterolemia was significantly increased in PNS patients [odds ratio (OR) = 6.40, 95% CI 2.06–19.87, *p* = 0.001] (Table 2). The AUC for the prediction of high levels of LDL-C in NS patients using plasma PCSK9 was 0.66 (95% CI 0.52–0.81, *p* = 0.047) (Figure 4(b)), the Youden index was 0.34, and the corresponding PCSK9 value was 255.05 ng/ml. The sensitivity of PCSK9 in predicting high levels of LDL-C was 0.77, and the specificity was 0.57. Logistic regression analysis showed that when plasma PCSK9 was >255.05 ng/ml, the risk of developing high levels of LDL-C was significantly increased in PNS patients (OR = 3.83, 95% CI 1.25–11.68, *p* = 0.018) (Table 2).

**Table 2.** The effect of plasma PCSK9 levels on hyperlipidemia in PNS patients.

	TC		LDL-C			
	OR *(95%CI)*	*p*-value			OR *(95%CI)*	*p* Value
PCSK9 ≤ 267.60	ref		PCSK9 ≤ 255.05		ref	
PCSK9 > 267.60	6.40 *(2.06–19.87)*	0.001	PCSK9 > 255.05		3.83 *(1.25–11.68)*	0.018

Abbreviations: PNS: primary nephrotic syndrome; PCSK9: proprotein convertase subtilisin/kexin type 9; OR: odds ratio; CI: confidence interval; TC: total cholesterol; LDL-C: low-density lipoprotein cholesterol.

### Expression of PCSK9 in patients with different pathological types of PNS

**Figure 5.**
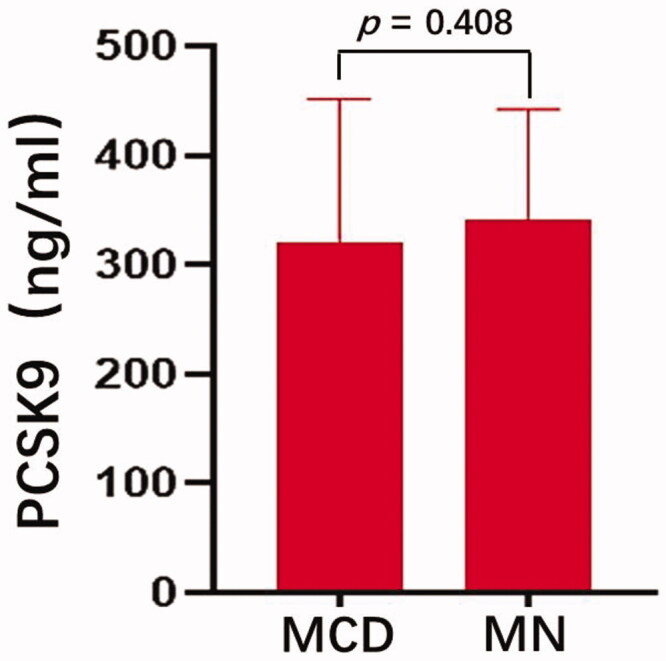
Comparison of plasma PCSK9 levels in patients with MCD and MN The plasma levels of PCSK9 in the MCD and MN groups were 320.51 ± 131.15 ng/ml 341.55 ± 100.54. ngmL, respectively, (*p* = 0.408). MCD, minimal change disease; MN, membranous nephropathy.

The main pathological types of PNS in this study were minimal change disease (MCD) and membranous nephropathy (MN), with 24 (21%) and 78 (67%) cases, respectively (Table 3). There were fewer patients with other pathological types. Therefore, differences in plasma PCSK9 levels were compared between patients with these two pathological types of PNS (MCD and MN groups). As shown in Figure 5, the plasma levels of PCSK9 in the MCD and MN groups were 320.51 ± 131.15 ng/ml and 341.55 ± 100.54 ng/ml, respectively, and this difference was not statistically significant (*p* = 0.408, Figure 5).

**Table 3.** Renal pathology of patients with PNS.

Pathological classification	*n* (%)
IgA nephropathy	7 (6%)
Minimal change disease	24 (21%)
Membranous nephropathy	78 (67%)
Focal segmental glomerulosclerosis	4 (3.75%)
Membranous proliferative glomerulonephritis	2 (1.5%)
Endocapillary proliferative glomerulonephritis	1 (0.75%)
Total	116 (100%)

## Discussion

PCSK9 is a serine protease that is produced and released into the circulation by the liver, and to a lesser extent by the intestine and kidney. Several studies have demonstrated significant direct correlations between plasma PCSK9 and LDL-C levels in the general population [17–19]. The present study demonstrated that plasma a healthy control group, and that elevated plasma PCSK9 levels had a positive linear correlation with elevated serum TC and LDL-C, which are the hallmarks of NS. Furthermore, we found that when PCSK9 was >255.05 ng/ml, NS patients were more prone to develop hypercholesterolemia. A comparison of plasma PCSK9 levels of MCD and MN patients showed that they were not significantly different.

Previous studies in animals found that NS models suffer from marked elevation of serum TC and LDL-C, which is accompanied by marked upregulation of hepatic PCSK9 expression [11,20]. Moreover, a cross-sectional study found a marked increase in plasma PCSK9 levels in nephrotic patients in whom plasma PCSK9 levels are directly related to TC and LDL-C concentrations [21]. Another longitudinal study found that patients with NS show decreased levels of plasma cholesterol and plasma PCSK9 in remission of their disease; in addition, changes in PCSK9 are correlated with changes in TC and LDL-C in NS remission [20]. These findings imply a consistent association between NS-associated hypercholesterolemia and PCSK9 in humans. However, only a small number of participants were involved in the above studies [20,21], and one-third of the patients were already undergoing immunosuppressive therapy to treat NS in the latter study [20]. We recruited 116 patients who suffered from nephrotic-range proteinuria, but who had normal renal function without treatment with statins or immunosuppressants. Consistent with previous studies [11,20,21], we found that elevated plasma PCSK9 levels in newly diagnosed PNS patients were linearly positively correlated with TC and LDL-C abundance. These findings suggested that PCSK9 may play important roles in NS-associated hypercholesterolemia.

Previously, the mechanism underlying hyperlipidemia in NS was considered to be a hypoproteinemia-induced increase in the compensatory synthesis of lipoproteins in the liver. Several studies have suggested that this is not the main mechanism and that LDL-R deficiency plays a more important role in hypercholesterolemia and elevation of plasma LDL-C in NS [3,5,6]. Earlier studies have demonstrated that circulating PCSK9 binds to LDL-R on the surface of hepatocytes, causing the receptor to be internalized and degraded in the lysosome [13]. Moreover, a study conducted in rats with NS found a significant reduction in hepatic LDL-R, accompanied by marked upregulation of hepatic PCSK9 expression, explaining why the receptor is depleted in NS [11]. By mediating the degradation of LDL-R, elevated plasma PCSK9 may play a major role in the pathogenesis of hypercholesterolemia in PNS patients. These findings suggest that PCSK9 may emerge as a novel therapeutic target for the treatment of NS-associated hypercholesterolemia.

PCSK9 exists in the form of monomers, dimers, and trimers in plasma, and the monomeric form of PCSK9 has very low LDL-R degradation activity. A previous study found that HDL-C regulates blood lipid levels by inhibiting PCSK9 self-association [22]; that is, a higher HDL-C level corresponds to greater PCSK9 monomer levels and less LDL-R degradation, which is conducive to cholesterol clearance. In the present study, we found that PCSK9 abundance was linearly positively correlated with HDL-C levels. Fan et al. also confirmed a positive correlation between PCSK9 and HDL-C levels in transgenic mice [22]. This evidence suggests that HDL-C may upregulate the proportion of PCSK9 monomers in plasma, and reduce its ability to degrade LDL-R, which is conducive to reducing blood lipid levels in PNS patients.

Haas et al. found that a ‘liver-kidney axis’ may exist in an NS mouse model, suggesting that kidney injury triggers an increase in the secretion of PCSK9 by the liver [20]. However, the molecular regulatory mechanisms involved in PCSK9 expression remain largely unknown in NS. Several studies have suggested that PCSK9 expression is regulated primarily by hepatocyte nuclear factor 1α (HNF-1α) and sterol regulatory element-binding protein 2 (SREBP-2) [23,24], and SREBP-2 is activated when intracellular free cholesterol levels decline. Studies by Vaziri et al. showed that hepatic acyl-coenzyme A cholesterol acyltransferase (ACAT), which catalyzes the intracellular esterification of cholesterol and thereby reduces intracellular free cholesterol levels, is upregulated in rats with puromycin-induced NS [25,26]. Moreover, up-regulation of hepatic ACAT is primarily due to proteinuria and not hypoalbuminemia [26]. Administration of an ACAT inhibitor to animals with NS accounts for dramatic amelioration of hypercholesterolemia, and a marked reduction in plasma LDL-C levels [25]. Therefore, we hypothesize that proteinuria may trigger the upregulation of hepatic ACAT and reduce the concentration of free cholesterol in hepatocytes, thus promoting the activation of SREBP-2 and upregulation of PCSK9 expression.

Statins are the cornerstone of current lipid-lowering therapy, and are beneficial for the induction of NS remission and for delaying renal disease progression [27–29]. However, in some patients administered the maximum tolerated dose of statins, plasma cholesterol levels still do not meet the desired standard. This situation is related to the lipid-lowering mechanism of statins. Statins inhibit cholesterol synthesis by inhibiting β-hydroxy β-methylglutaryl-CoA (HMG-CoA), resulting in a decrease in intracellular free cholesterol and activation of SREBP2. Therefore, the application of statins can elevate plasma PCSK9 levels, which may explain why some patients do not achieve ideal blood lipid levels after the application of statins. Careskey et al. found that the application of PCSK9 inhibitors, in addition to statins, enhances the lipid-lowering efficacy of statins [30]. In addition, Haas et al. found that PCSK9 ablation can reduce plasma triglycerides and cholesterol levels by 40% to 60% in mice with NS [20]. Recently, Morino et al. reported a case of renal cholesterol crystal embolism (CCE) induced by carotid artery stenting that was successfully treated with Evolocumab, a fully human monoclonal antibody against PCSK9, suggesting that inhibition of PCSK9 may have a beneficial effect in terms of renal involvement in patients with CCE [31]. Our study found that when plasma PCSK9 abundance in NS patients was > 267.60 ng/ml, the risk of developing hypercholesterolemia was significantly increased (OR = 6.40, 95%CI 2.06–19.87, *p* = 0.001); when plasma PCSK9 in NS patients was > 255.05 ng/ml, the risk of developing type II hyperlipoproteinemia was significantly increased (OR = 3.83, 95%CI 1.25–11.68, *p* = 0.018). Therefore, we speculate that for PNS patients, when levels are increased to 255.05 ng/ml, PCSK9 participates in the development of hyperlipidemia. The above data suggest that if the effect of statin therapy is not satisfactory, PCSK9 inhibitors might benefit PNS patients in treating NS-associated hypercholesterolemia.

In this study, 116 patients with PNS were enrolled, and the main pathological PNS types were MCD and MN. A comparison of plasma PCSK9 levels of MCD and MN patients showed that the difference was not statistically significant. Pathologically, MCD showed extensive foot process fusion in podocytes, vacuolar degeneration in epithelial cells, and microvilli degeneration. MN is caused by the diffuse deposition of immune complexes in glomerular epithelial cells. Under an electron microscope, there are neatly arranged, electron-dense substances on the epithelial side of the glomerular basement membrane (GBM) in the early stage of MN, and these substances are often accompanied by extensive foot process fusion. Haas et al. proposed a theory of podocyte-hepatocyte intersection, suggesting that podocyte injury can induce increased PCSK9 levels [20]. Both the MCD and MN groups exhibited podocyte injury, and there was no significant difference in plasma PCSK9 levels between the two groups. The expression of PCSK9 in other pathological types needs to be further explored in studies with larger sample sizes.

In summary, PCSK9 expression levels in newly diagnosed PNS patients were significantly higher than those in healthy controls, and linearly positively correlated with TC and LDL-C abundance, suggesting that PCSK9 is involved in the development of hyperlipidemia in PNS. Furthermore, with PCSK9 > 255.05 ng/ml, patients with PNS were more prone to develop hyperlipidemia. These results suggested that the addition of PCSK9 inhibitors may be beneficial for such PNS patients in the treatment of hypercholesterolemia. This finding provides a theoretical basis for the clinical application of PCSK9 inhibitors in PNS patients with hypercholesterolemia.

The subjects of this study were newly diagnosed with PNS, and the influence of confounding factors such as diabetes and lipid-lowering drugs on the expression of PCSK9 were excluded. Compared with those of previous studies, the results of the present study reflect the initial expression levels of PCSK9 in patients with PNS. However, this was a single-center, cross-sectional study; multi-center, prospective studies are needed to confirm the results of this study.
